# Neuropsychological Correlates of Linguistic Skills in At-Risk and Typically Developing Readers Across Educational Stages

**DOI:** 10.3390/brainsci16050442

**Published:** 2026-04-22

**Authors:** Inmaculada Méndez-Freije, Débora Areces, Celestino Rodríguez

**Affiliations:** Department of Psychology, University of Oviedo, Plaza de Feijoo s/n, 33003 Oviedo, Spain; mendezinmaculada@uniovi.es (I.M.-F.); rodriguezcelestino@uniovi.es (C.R.)

**Keywords:** reading difficulties, reading, morphological awareness, early detection, linguistic variables

## Abstract

**Highlights:**

**What are the main findings?**

- Differences in morphological skills and Oral Morphological Awareness relate mainly to diagnosis and school year.
- Children’s oral morphology performance may be an effective screening for RD.

**What are the implications of the main findings?**

- The type and frequency of oral morphological errors suggest that students at risk should be screened early on.
- Orally assessing Morphological Awareness may help identify children at risk before their formal reading skills do.

**Abstract:**

**Background:** Reading is a fundamental skill for children’s cognitive, social, and academic development which relies on the integration of multiple linguistic and cognitive abilities. Longitudinal studies consistently show that oral language skills predict reading development both in typically developing children and in those at risk for reading difficulties (RD). Despite strong empirical evidence, a gap remains between research and educational practice. **Objective:** The present study aims to compare linguistic variables, including vocabulary, oral text comprehension, oral morphological awareness (OMA), and morphological skills between diagnostic group (control vs. at-risk), study grade, and sex. **Method**: The study included 93 Spanish-speaking children aged 6 to 12 years (*M* = 8.7, *SD* = 1.9; 50 boys, 43 girls). Two diagnostic groups were established: 44 children at risk of reading difficulties (including ADHD or DLD) and 49 typically developing controls. Participants were also classified by academic cycle: 55 in the first cycle (1st–2nd grade) and 38 in the second cycle or higher (3rd–6th grade). Linguistic variables were measured through tests administered individually, with data collected during one-on-one assessment sessions. **Results:** Among the variables analysed, significant differences were observed only in morphological skills and OMA. No significant differences were found based on sex, whereas both academic cycle and diagnostic group showed significant effects. **Conclusions:** The most relevant and novel finding is that the type and frequency of errors in the OMA task could serve as an early indicator of students at risk of RD. OMA assessment could therefore be a promising method of early screening and targeted interventions.

## 1. Introduction

Reading is a fundamental skill for cognitive, social, and academic development. In addition to its acting as a tool for accessing information, reading facilitates the acquisition of knowledge, the development of critical thinking and people’s engagement in cultural and social life [1]. In a school setting, learning to read is considered a milestone in children’s academic development, since reading has a cross-cutting impact on all areas of the curriculum, so the capacity to read proficiently is a critical factor in achieving academic success. But learning to read is a complex cognitive skill that requires the integration of multiple cognitive [2,3] and linguistic skills [4,5]. Key linguistic components include phonological awareness (PA), the alphabetic principle (AP), morphological skills, vocabulary size, and listening comprehension [6,7]. At the cognitive level, processing speed influences both sublexical decoding and lexical access, while working memory (WM) plays a critical role in the short-term integration and storage of phonological, orthographic, and semantic information. Additionally, the allocation of cognitive resources and the coordination of lower- and higher-level reading processes are influenced by executive functions such as attentional control, cognitive flexibility, and inhibition [2,3]. These cognitive and linguistic processes are impaired across several neurodevelopmental disorders, including attention-deficit/hyperactivity disorder (ADHD), dyslexia, and developmental language disorder (DLD). Despite the considerable variability and heterogeneity of these conditions, they are characterized by a high degree of comorbidity and substantial overlap in linguistic and cognitive deficits [8,9], which in turn results in a high prevalence of reading difficulties (RD) in these populations [8,10].

In light of this, a transdiagnostic approach may offer a useful framework for understanding reading difficulties (RD), not simply as the result of disorder-specific deficits, but as the outcome of dynamic interactions among multiple risk and protective factors distributed along a continuum [11].

Although there is extensive and solid scientific evidence on learning to read, there is a significant gap between research and educational practice. This often results in students failing to achieve the required literacy levels, particularly those who exhibit linguistic deficiencies or originate from socio-economically disadvantaged environments [1].

A substantial body of longitudinal research has consistently demonstrated a significant relationship between oral language skills and reading ability. For instance, research carried out by Hulme et al. [12], which involved children with pre-school language difficulties, children at risk of dyslexia and children with typical development, showed that early oral language skills reliably predict subsequent reading ability. The researchers found that measures of oral language in preschool children predicted phoneme awareness and grapheme–phoneme knowledge shortly before school entry. These, in turn, predicted word-level literacy skills shortly after school entry. These results highlight the fact that early oral language skills are reliable predictors of subsequent word-level literacy and reading comprehension ability in both typically developing children [13,14,15] and those at risk of literacy difficulties [16,17].

The findings of the longitudinal study conducted by Van Viersen et al. [16] are along the same lines. The results of this study showed that there are two paths to reading comprehension: one mediated by decoding skills and the other based on early language skills, without going through decoding. Therefore, at this developmental stage, early oral language is associated with both word decoding and comprehension.

These longitudinal studies show that the predictive links between oral language and reading development are evident in both typically developing readers and children at risk of reading difficulties (RD) [12,14,16,18]. These results are highly relevant in clinical and educational settings [19]. Longitudinal research also demonstrated that there are direct and indirect predictive pathways through which specific components of oral language, such as vocabulary, morphosyntactic knowledge and phonological awareness, influence reading acquisition over time [13,17].

Nevertheless, the relationship between oral language skills and early reading outcomes is intricate, as it can fluctuate based on the specific language skills involved and contextual factors [16,20].

From an educational perspective, oral language is regarded as a fundamental skill that can act as a predictor of reading acquisition, but it is also considered a strong predictor of academic outcomes and success in life [4]. Therefore, the systematic assessment of oral language in educational settings can facilitate the early identification of difficulties and facilitate the development of specific interventions. Furthermore, structured language support can be beneficial even for students in the final years of primary education, as demonstrated by a recent study conducted by Esposito et al. [21]. This study, despite certain limitations, showed that after the implementation of the Oral Language for Literacy Intervention (OLLI) programme, there were significant improvements in written expression and oral language in children aged 8 to 9 years.

Although oral language plays a fundamental role in literacy development, it is important to recognise that reading differs from oral language in that it is not an innate human ability, but rather a skill that is passed down through culture and must be explicitly taught. In reality, reading and oral language are not entirely independent processes; rather, reading builds upon the neural and linguistic systems that individuals develop for processing spoken language and gradually reshapes them over time [22].

From a cognitive neuroscience perspective, the neurological mechanisms of reading rely on an interactive brain network, located mainly in the left hemisphere, which enables the transformation of visual information into orthographic, phonological and semantic representations [23,24]. This network extends to temporo-parietal and frontal language systems in addition to occipito-temporal regions, such as the visual word form area. A substantial amount of data points to a dual-stream arrangement, with a dorsal channel involved in phonological processing and grapheme-to-phoneme conversion and a ventral pathway mostly linked to visual-orthographic processing and lexical-semantic access [24,25]. The interplay between these routes makes flexible and context-dependent reading possible. These pathways interact dynamically, rather than functioning as a rigidly hierarchical system, and involve temporo-parietal regions associated with phonological processing and frontal areas linked to articulation, executive control and verbal working memory [24,25,26]. Finally, brain plasticity enables the brain circuits responsible for visual language functions to adapt to the demands of literacy culture without suppressing their previous functions [22,27,28]. Taken together, these neurocognitive mechanisms may help explain how reading is acquired and taught.

In alphabetic languages with relatively transparent orthographies, such as Spanish, a central instructional goal is to grasp the AP, or the idea that graphemes systematically correspond to phonemes in spoken language [29,30]. Mastery of these grapheme–phoneme correspondences forms the basis of decoding and encoding processes in reading and writing.

To understand the AP, it is essential to master phonemic awareness: a knowledge of the orthographic symbols (letters), their names and their associated sounds. It is important to note that these skills interact bidirectionally, i.e., explicit teaching of the correspondence between letters and sounds is not only based on phonemic awareness but also reinforces it, thus promoting dynamic early literacy development [1,31,32].

PA, a crucial skill, is concerned with the recognition and manipulation of the sound units in spoken languages. This important ability is crucial when developing decoding abilities. Early exposure to, and comfort with, phonological structure eases word recognition, directly assisting in the evolution toward proficient, comprehensible reading. Conventionally, PA has been widely accepted to be a very strong indicator of how reading skills develop [1,33,34]. Despite this, emerging research throws a slightly different light on the situation, indicating that the predictive power of PA may not be universal; in some studies, other pre-literacy skills show equal or greater predictive value, especially when contextual factors such as orthographic transparency are taken into account [35,36,37].

Even though it is well established that the AP and PA are fundamental to efficient word recognition, particularly with transparent orthographies, we should not only focus on reading accuracy but also take reading fluency and comprehension into consideration. Both of these aspects are based on linguistic skills such as vocabulary, morphosyntactic knowledge and morphological awareness and they determine reading achievement far beyond the initial stages of decoding [14,32,33,36]. Basic word decoding skills provide the starting point for reading, but the ongoing development of reading depends on the integration of different elements of language and that may vary depending on the spelling system each language presents [15,38,39]. In more advanced stages, reading performance is increasingly supported by the coordinated contribution of higher-order linguistic systems, particularly those related to vocabulary, morphosyntax, and morphological processing, which facilitate fluent and meaningful text comprehension. Overall, the reviewed evidence suggests that reading development is best conceptualized as a dynamic and cumulative process, whereby early decoding skills provide the initial entry into print, while sustained proficiency emerges from the progressive integration of multiple interacting language domains.

The Simple View of Reading (SVR), initially proposed by Gough and Tunmer in 1986 [40], is a widely used model to explain the development of reading skills to teachers and other professionals. According to the SVR model, reading is the product of two independent components: decoding (D) and listening comprehension (C), which is captured in the following equation: D × C = R. Based on this model, three types of reading disabilities can be identified: difficulties with decoding, which the authors called dyslexia; difficulties with listening comprehension, which they called hyperlexia, and difficulties in both components, which they referred to as garden-variety reading disability [40,41].

Decoding skills, like listening comprehension skills, vary throughout the learning to read process. At first, decoding is essential for reading comprehension, but once decoding becomes automatic, listening comprehension becomes essential for understanding what is being read [40,42,43].

Although the SVR model greatly simplifies the picture for understanding RD in children, not all RD are limited to problems at the level of decoding and/or auditory comprehension. Furthermore, there are other factors that can significantly influence reading. In response to these limitations, the Active View of Reading Model has recently emerged as an extension of the SVR model which includes self-regulatory and motivational cognitive processes [44,45].

There is also robust evidence that oral comprehension, vocabulary and morphological skills are indispensable linguistic factors within literacy development because they together explain a considerable segment of reading comprehension variation and provide unique and well complementary contributions to the development of reading skills [15,18,38,46].

Regarding morphological awareness (MA), commonly defined as the ability to reflect upon and manipulate the morphemic structure of words, it has been shown to be robustly associated with reading skills such as word reading, spelling, and reading comprehension [47,48,49,50]. Despite strong empirical evidence supporting this relationship, the specific roles of MA and morphology have historically been underestimated in theoretical models of reading development. In this sense, the Morphological Pathways Framework [51] provides a comprehensive account of the intricate relationship between morphological knowledge and reading performance, including both direct and indirect pathways. This framework extends the established Reading Systems Framework [52] and addresses previous gaps by specifying the multiple ways in which morphology supports word reading, spelling, and reading comprehension. It is therefore an invaluable resource for researchers and educators seeking to enhance literacy development through morphological instruction.

Although there are some standardised tests in English for assessing MA (e.g., the Morphological Awareness Test for Reading and Spelling (MATRS) [53] or subtests like the Test of Language Development–Primary, Fourth Edition (TOLD-P:4) [54]), clinical reviews indicate that there is currently no instrument available that provides a comprehensive assessment of the MA [55,56,57]. For this reason, MA is still predominantly assessed using well-designed, but no standardized, researcher-created measures. In these cases, both the type of task and the characteristics of the morphemes (type of morpheme, frequency, imageability, etc.) must be carefully selected. The tasks must also be adapted according to the age of the children and the specific assessment objective. In addition, MA is a multidimensional construct. Consequently, current assessment instruments often differentiate between diverse types of morphology, such as inflectional, derivational, and compositional, as well as between various dimensions of morphological processing that encompass semantic, syntactic, and orthographic or phonological components. It is also recognised that MA in the broad sense, morphological decoding, and morphological analysis are closely related but conceptually distinct skills that play partially differentiated roles in language development and reading acquisition [58,59,60].

On another note, a substantial proportion of research on the acquisition of reading skills has been conducted on their acquisition in English, which may represent a significant limitation for a universal science of reading. Generalizing results based on a language with a complex and non-transparent spelling system, such as English, could be skewing our understanding of reading development and learning to read in languages with transparent orthographies. This is because theoretical models have been developed that may not be fully applicable to languages with transparent orthographies [61,62].

For all of the above reasons, it is particularly important to examine the extent to which the different elements of oral language interact with reading in languages with a transparent spelling, such as Spanish. Few studies conducted in Spanish have adopted a comprehensive approach to linguistic skills across different neurodevelopmental disorders while simultaneously considering early predictors of reading that are independent of reading performance.

The objective of the present study is to compare linguistic variables, such as vocabulary level, oral text comprehension, oral morphological awareness, and morphological ability, according to diagnostic group (control vs. at-risk), study cycle, and sex, with the aim of providing findings with both broad and educationally relevant implications. Linguistic variables will also be examined as a function of sex. Previous studies have investigated sex differences in linguistic abilities, with some evidence suggesting a slight female advantage. However, these differences tend to diminish with age, as other factors such as educational level, socioeconomic background, and individual strategies, gain greater explanatory power [58,59,60,61,62,63,64]. Notably, these studies have not included participants with neurodevelopmental disorders.

## 2. Materials and Methods

### 2.1. Participants

A total of 93 students aged 6–12 years (*M* = 105.24 months; *SD* = 19.16) participated, of whom 50 were boys and 43 were girls. The mean age of the boys was 103.90 months (*SD* = 20.36) and the mean age of the girls was 106.79 months (*SD* = 17.78).

This study employed a transdiagnostic approach to address the heterogeneity inherent in neurodevelopmental disorders and the presence of shared risk factors [11]. Accordingly, the total sample was divided into two groups: a group of 44 students at risk of RD (*M_age(months)_* = 116.09; *SD_age(months)_* = 21.72), including participants with a previous diagnosis of ADHD or DLD, and a control group (*M_age(months)_* = 95.49; *SD_age(months)_* = 8.71) consisting of 49 participants without a diagnosis (neurotypical group).

Two groups were formed according to study cycle in order to account not only for age but also for the academic skills associated with each educational stage, so that the results may have meaningful implications for educational practice: the ‘first cycle’ group (1st and 2nd year of primary education) consisted of 55 students whose mean age was 92.91 months (*SD* = 5.24), and 38 participants formed the ‘second cycle or higher’ group (students from 3rd to 6th year of primary education), whose mean age was 123.08 months (*SD* = 17.89).

All participants were native Spanish speakers with normal or corrected-to-normal vision. They were recruited from various public primary schools, speech and language therapy clinics, and psychoeducational centres in the Principality of Asturias, in Spain. Participants were excluded if they had been diagnosed with a neurodevelopmental disorder other than ADHD or DLD, with hearing loss, deafness, an intellectual disability, or had a history of neurological disease or epilepsy. Inclusion in the group of students at risk of RD was based on official reports of a recognized developmental disorder (including dyslexia, ADHD, and/or DLD) or a specific learning difficulty in reading, as determined by psychologists at the respective clinics or school guidance departments; in all cases, participants with these neurodevelopmental disorders were required to present reading difficulties as a common symptom.

### 2.2. Procedure

The study was approved by the Research Ethics Committee of the University of Oviedo on 22 May 2023 (reference code: 18_RRI_2023). Active informed consent was signed by the parents or legal guardians of each participant prior to data collection.

To achieve the objectives of this study, all participants were tested individually in a quiet room with no distractions. The assessment included tests of receptive vocabulary, oral text comprehension, morphosyntactic ability, and Oral Morphological Awareness (OMA). All tests were administered in a fixed order: receptive vocabulary was assessed first, followed by oral text comprehension. A short break was then introduced to reduce participant fatigue. OMA was subsequently evaluated, and morphosyntactic ability was assessed last.

### 2.3. Instruments and Variables

#### 2.3.1. Receptive Vocabulary

The PEABODY Picture Vocabulary Test (PPVT-III) [65] was administered to measure receptive vocabulary. This test, which is suitable for subjects from 2 to 90 years of age, contains 192 sheets, each with four pictures. The Spanish version of the PPVT-III was used, which involved a cross-cultural translation and adaptation process in which items were linguistically and culturally adjusted to ensure semantic equivalence and familiarity across Spanish-speaking populations. The resulting version was normed on large samples of Spanish-speaking children from Spain and several Latin American countries, and standardized scores were derived through age-based norming procedures. The subject must indicate which picture best represents the meaning of the word given by the evaluator. Cronbach’s alpha for the Peabody test in this study was 0.71.

#### 2.3.2. Oral Comprehension of Texts

Oral comprehension was assessed using the Text Comprehension Task from the fifth edition of the Clinical Evaluation of Language Fundamentals (CELF-5) [63]. In this task, participants must answer questions about a text read aloud by the evaluator. The questions focus on the text’s main idea, as well as other deductive and predictive information. The reliability of this test in the study sample was Cronbach’s α = 0.96. The Spanish version of the test was used, which was normed on a representative sample of the Spanish population comprising 922 participants, with stratification based on sex, age, and educational level.

#### 2.3.3. Morphosyntactic Ability

Morphosyntactic ability was assessed using the morphosyntactic task in the CELF-5 battery [63]. In this task, participants observe pictures in a stimulus booklet and must complete a read-aloud sentence as appropriate. Cronbach’s alpha for this task was 0.89 in the study sample. The Spanish standardized version of the test was used, based on a representative sample of 922 participants from the Spanish population, stratified by sex, age, and educational level [63].

#### 2.3.4. Oral Morphological Awareness (OMA)

**Figure 1:**
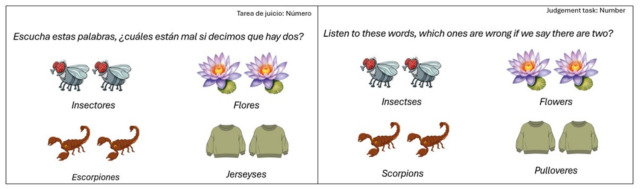
Example of a Judgement task in Spanish and English.

**Figure 2:**
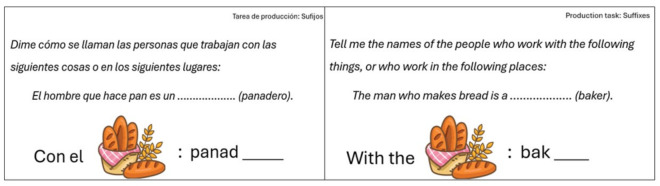
Example of Production task in Spanish and English.

The IECMO test (in Spanish, Instrumento de Evaluación de la Conciencia Morfológica Oral [Oral Morphological Awareness Test]) [64] was used to assess Oral Morphological Awareness (OMA) in Spanish. The IECMO test considers inflectional morphology, derivational morphology and compounding morphology. The test is administered individually and orally. It consists of a total of 60 items, 30 of which correspond to judgement tasks and 30 to production tasks (Figure 1 and Figure 2). The IECMO produces a dual score: the total number of correct scores and the total number of errors made in the two levels being assessed (morphological production and morphological judgement). In addition, it is possible to obtain the total OMA score, which is the sum of the scores in both levels. The analysis of the internal consistency reliability of the test yielded Cronbach’s alpha values of 0.91 and 0.86 in the study sample.

The independent variables in this study were group, sex, and cycle of studies, while the dependent variables were the percentile scores for receptive vocabulary, oral comprehension of texts, and morphosyntactic ability, as well as the raw score for errors in the OMA task (total score of errors in inflectional morphology, derivational morphology and compositional morphology).

#### 2.3.5. Data Analysis

To achieve the objectives of this study, various statistical analyses were performed using SPSS version 27. Firstly, Analyses of Covariance (ANCOVAs) were performed to evaluate differences in vocabulary level, oral comprehension and morphosyntactic ability according to group (control vs. at-risk), cycle (first cycle vs. higher cycles) and sex.

Subsequently, three Multivariate Analyses of Covariance (MANCOVAs) were performed to analyse the differences in OMA according to the three independent variables of this study (sex, cycle and group), considering the effect of the other independent variables in each case. Three dependent variables related to OMA were considered: errors in inflectional morphology, derivational morphology, and compositional morphology. Preliminary assumption testing was conducted in all of the analyses to check for normality, linearity, univariate and multivariate outliers, homogeneity of variance-covariance matrices, and multicollinearity. No serious violations were noted.

## 3. Results

### 3.1. Preliminary Analysis

First, descriptive statistics (Table 1) were calculated to verify that all variables were approximately normally distributed according to Kline’s criterion [66]. And the results indicated the possibility to carry out parametric analysis.

### 3.2. Differences in Linguistic Variables as a Function of a Group (Control Group vs. At-Risk Group)

#### 3.2.1. Vocabulary

A one-way analysis of covariance (ANCOVA) was conducted to compare vocabulary levels between the control group and the group at risk of RD. The group was the independent variable, and the dependent variable was the percentile score on vocabulary. The cycle of study and sex variables were used as covariates in this analysis. No statistically significant differences were observed (*F* (1, 89) = 0.275, *p* = 0.602).

#### 3.2.2. Oral Comprehension

A MANCOVA was performed to examine the effect of group (control vs. at-risk) on text comprehension levels, while controlling for the influence of the variables cycle of study and sex. No statistically significant differences were found between participants according to group (*F* (1, 89) = 0.290, *p* = 0.592).

#### 3.2.3. Morphosyntactic Ability

An ANCOVA was performed to analyse whether there were statistically significant differences in morphosyntactic ability between the control group and the group at risk of RD. In this analysis, the dependent variable was the percentile score for morphosyntactic ability; the group was the independent variable; and the cycle of study and sex were used as covariates.

The results showed statistically significant differences in morphosyntactic ability [*F* (1, 89) = 17.36, *p* < 0.001, η_p_^2^ = 0.163]. On average, participants in the control group ranked higher in percentile (*M* = 77.41, *SD* = 16.41) than participants at risk of RD (*M* = 58.75, *SD* = 22.99).

#### 3.2.4. Errors in OMA Task

A MANCOVA was performed to investigate differences in OMA between the control group and the group at risk of LD (Table 2). The analysis controlled for the effects of the study cycle and sex.

Significant differences were observed between the groups in the number of errors in the OMA task [*F* (3, 87) = 13.17, *p* < 0.001*; λ* = 0.688; *η_p_*^2^ = 0.312]. The results of the inter-subject effects tests revealed significant differences between the two groups across the three types of OMA errors. The group at risk of RD made more errors in all cases.

### 3.3. The Effect of the Variable Studies Cycle

#### 3.3.1. Vocabulary

ANCOVA was performed to analyse whether there were differences in vocabulary levels as a function of study cycle, taking sex and group (control vs. at-risk) as covariates. No significant differences were found (*F* (1, 89) = 2.84, *p* = 0.096).

#### 3.3.2. Oral Comprehension

ANCOVA was performed to examine the effect of the variable cycle of studies (first cycle vs. higher cycles) on text comprehension levels, controlling the influence of the variables group (control vs. at-risk group) and sex. No statistically significant differences were found between participants according to the study cycle (*F* (1, 89) = 0.760, *p* = 0.386).

#### 3.3.3. Morphosyntactic Ability

To evaluate whether there were differences in morphosyntactic ability between participants in the first cycle of studies and those in higher cycles, an ANCOVA was performed, controlling for the effects of sex and group on the dependent variable (morphological ability percentile score) no statistically significant differences were observed (*F* (1, 89) = 3.02, *p* = 0.085).

#### 3.3.4. Errors in OMA Task

A MANCOVA was conducted considering the effect of the others independent variables (sex and group) to determine whether there were differences in the number of morphological errors (errors in inflectional morphology, derivational morphology and compositional morphology) in the OMA task based on the cycle of study. The results showed significant differences in the number of morphological errors depending on the participants’ cycle of study [*F* (3, 87) = 14.58, *p* < 0.001; *λ* = 0.665; *η_p_*^2^ = 0.335].

The results of the inter-subject effect tests showed significant differences in the number of each of the three types of morphological error between participants in the first and higher cycles (Table 3). In general, participants in the first cycle made more errors than those in higher cycles. Most of these errors were derivational morphology errors, followed by inflectional morphology errors.

### 3.4. The Effect of the Sex Variable

#### 3.4.1. Vocabulary

ANCOVA analyses were calculated to assess whether there were differences in participants’ vocabulary levels according to gender. The group (control vs. at-risk) and the study cycle (first vs. higher) were used as covariates. No statistically significant differences were observed (*F* (1, 89) = 0.460, *p* = 0.499).

#### 3.4.2. Oral Comprehension

A MANCOVA was performed to examine the effect of the variable sex on text comprehension levels, while controlling for the influence of the variables group (control vs. at-risk group) and cycle of studies (first cycle vs. higher cycles). No statistically significant differences were found between participants according to the study cycle (*F* (1, 89) = 0.156, *p* = 0.694).

#### 3.4.3. Morphosyntactic Ability

No statistically significant differences were observed (*F* (1, 89) = 0.030, *p* = 0.863) in the ANCOVA carried out to analyse the effect of sex on morphosyntactic ability.

#### 3.4.4. Errors in OMA Task

MANCOVA was performed to assess differences in the number of errors made in the OMA task and the effect of the other independent variables (sex and group) was considered. The results showed no significant differences between participants according to gender (*F* (1, 87) = 1.857, *p* = 0.143).

## 4. Discussion

The purpose of this study was to compare variables related to oral language, such as receptive vocabulary level, oral comprehension, morphological ability, and OMA of participants according to sex, study cycle, and diagnostic group (control vs. at-risk).

Several recent studies have revealed significant differences in morphological skills between typical and at-risk readers and their reading comprehension, highlighting the need to examine how these associations vary by reader profile [49,67,68,69]. In addition to morphological ability and OMA, other linguistic variables have also been shown to differentiate typical and at-risk readers. For example, readers at risk of RD tend to exhibit weaker oral language skills than typically developing readers, particularly in receptive vocabulary, grammatical comprehension, and communicative abilities, even when basic decoding skills are relatively preserved [70,71]. However, among all the variables analysed in the present study, significant differences were found only in morphological ability and OMA. The findings did not show significant differences in the variables based on sex, but they did show differences based on study cycle and diagnostic group.

Although some research has reported a slight advantage for females in certain verbal tasks [72,73], the present study revealed no consistent sex differences in the linguistic variables measured. The findings corroborate earlier research demonstrating that educational background and linguistic experience, along with the quality of instruction, can reduce or eliminate gender discrepancies, particularly when both genders have similar opportunities for education and linguistic exposure [74,75]. This affects skills such as receptive vocabulary, oral comprehension and MA [76,77]. Therefore, the absence of sex-related differences suggests that biological sex and gender-related factors alone do not determine performance in the analysed linguistic variables. Previous research suggests that although minor sex- or gender-related differences in linguistic performance may appear in certain contexts, such differences are neither consistent across studies nor sufficient to account for overall linguistic outcomes. Instead, outcomes emerge from the interaction of diverse factors such as age, task types, and sociocultural context, among others [72,78,79].

In the present study, statistically significant differences were observed in the number of errors in the OMA task depending on the study cycle. In general, participants in the first cycle made more errors than those in higher cycles across all three morphological error type (inflectional, derivational, and compounding).

From a developmental perspective, we can explain these differences with reference to the fact that morphological skills appear to emerge from cumulative exposure to morphologically complex language and are reinforced through literacy and oral interaction, rather than functioning as an isolated or purely innate ability. This aligns with the meta-analysis conducted by Bratlie et al. [80], which showed that children with limited exposure to the majority language display systematically lower performance on morphological tasks, especially those requiring oral production and explicit morphological manipulation. The reduced morphological competence observed in the at-risk group in the present study mirrors this pattern, highlighting the critical role of language exposure and oral practice in the development of morphological skills.

The differences observed in the present study are to be expected if we consider that morphological skills develop gradually throughout schooling [38,51]. The reduction in morphological errors in higher grades can therefore be explained by the progressive development of MA, together with cognitive maturation and greater exposure to educational experiences. It is likely that lower-grade pupils have not yet fully consolidated morphological rules and exceptions, whereas with greater exposure to written language, these structures begin to become automatic and errors become less frequent.

Overall, the results obtained are consistent with longitudinal research demonstrating that MA develops progressively and contributes to literacy outcomes [47,81]. Notably, these findings reinforce the notion that MA is a dynamic skill that can be improved through specific educational interventions, potentially supporting enhancements in reading and spelling proficiency [82].

Considering the diagnostic group (control vs. at-risk), statistically significant differences were observed between the two groups in terms of morphological ability and the number of errors produced in the OMA task. Participants in the control group performed better than those in the at-risk group, demonstrating higher levels of morphological competence and making fewer errors. These results are similar to those in previous studies documenting that children with dyslexia or RD often exhibit MA deficits across different languages [12,83,84].

MA can be assessed through oral, written, or combined modalities, with the choice of format depending on the population and the literacy outcomes of interest. Assessing MA has been shown to play a critical role in the early identification of language and literacy difficulties [85,86,87]. In this context, oral MA tasks are particularly valuable, as they allow for the assessment of emerging morphological skills while minimising confounds related to decoding and orthographic knowledge [56,88,89]. Consequently, oral MA measures are especially suitable for use in early diagnostic and research contexts, when children’s literacy skills are still developing, thereby opening up the possibility of incorporating oral MA tasks into early assessment batteries. Therefore, clinical guidelines recommend that speech–language pathologists and other professionals involved in early assessment consider oral, and when appropriate dynamic, MA tasks, selecting assessment formats based on children’s age, literacy level, and the specific aims of the evaluation [56].

Concurrently, previous research has underscored the significance of MA in reading development. Specifically, MA has been shown to account for unique variance in the word-reading ability of children over the age of eight, even when age, PA and naming speed are taken into account [49,90]. However, these group differences should be interpreted cautiously because the prevalence of MA deficits among children with dyslexia varies widely (12–76%), with a combined estimate of around 40%, highlighting individual variability and the influence of language, age, and assessment methods [15,91]. Furthermore, in their recent meta-analysis Georgiou et al. [67] reported that, while children with developmental dyslexia perform significantly worse than age-matched peers on MA tasks, these differences disappear when comparisons are made with younger children matched on reading level. This result suggests that low morphological competence may not be the main causal factor of RD, but rather a consequence or correlate of these difficulties. Taken together, these findings indicate that morphological skills should be considered a significant domain for supporting reading development.

In line with this, some studies have suggested that children with RD may use morphological information as a compensatory strategy when phonological processing is impaired. These studies have also found that morphological training can improve spelling and reading outcomes, which supports the idea that interventions targeting MA can enhance spelling and reading performance, supporting the potential effectiveness of such targeted training [68,92,93].

When we consider the types of errors observed in the OMA task in both the educational cycle and diagnostic group comparisons, we find that errors in derivational morphology were the most frequent, followed by errors in inflectional morphology. These results are to be expected, given that various studies conducted in Spanish-speaking contexts indicate that derivational morphology develops later and is more challenging than inflectional morphology. This difference is explained by the lower semantic transparency of derivational morphology and the greater complexity of the processes involved in transforming both the grammatical category and the meaning of words [94]. In particular, D’Alessio et al. [48] demonstrate that Spanish-speaking children process derivational morphemes more slowly than inflectional morphemes in word naming tasks. This highlights the greater cognitive demand associated with derivation. Similarly, studies involving participants with RD have revealed significant deficits in derivational tasks due to their greater cognitive demand and the lower semantic transparency of many derivations [83,84]. Furthermore, research with Spanish-speaking children suggests that MA, particularly derivational MA, is significantly associated with reading skills. This indicates that difficulties in this area may have a detrimental impact vocabulary expansion and reading comprehension [93]. Similarly, the meta-analysis by Bratlie et al. [80] shows that derivational morphology amplifies differences between groups with varying levels of linguistic competence, particularly in tasks involving orally mediated tasks. This is evident in the OMA task in our study, where morpheme manipulation is performed orally.

### Limitations

The results obtained suggest a basis for the development of early detection and personalised monitoring strategies, offering a promising starting point for preventive educational interventions and future research on morphological and linguistic development. However, it is important to note that research on this topic that is specific to Spanish language remains scarce. Furthermore, the reported findings exhibit significant variability depending on the type of task used to assess MA and the specific morphology examined. Some limitations of the present study also need to be considered, including the absence of specific standardised tests to assess MA in Spanish and the small sample size, which may limit the generalisability of the findings.

On the other hand, these results could be interpreted in light of the limitations of traditional categorical diagnostic frameworks, which may fail to adequately capture the heterogeneity of developmental profiles and the shared underlying mechanisms across different neurodevelopmental conditions. From a transdiagnostic perspective, focusing on common cognitive and linguistic processes, rather than on discrete diagnostic categories, may provide a more accurate account of the variability observed in reading-related outcomes [11]. For this reason, the present study adopts a transdiagnostic approach to identify early linguistic predictors in a population with various neurodevelopmental disorders, such as ADHD, dyslexia, and/or DLD, which share the presence of RD as a common symptom.

In view of these factors and the current scarcity of research, further investigation in this field is warranted.

## 5. Conclusions

The present study examined differences in linguistic variables according to diagnos-tic group, study cycle, and sex. The results indicated that meaningful differences were limited to morphological skills and OMA and were predominantly associated with the diagnostic group and the study cycle. It can therefore be concluded, as the relevant and innovative finding, that it could be possible to carry out an early screening of students at risk of RD based on the type of morphological error and the frequency of these errors in the OMA task. Notably, the analysis of the type and frequency of errors in the OMA task suggests that oral morphological performance may serve as an early indicator of risk for RD. Because this task can be administered orally, it allows for the identification of vulnerable children before formal reading skills are fully established, opening the door to earlier detection and more timely, targeted educational support.

## Figures and Tables

**Table 1:** Descriptive statistics of all variables.

Variable	*M*	*SD*	Min	Max	Skewness	Kurtosis
PEABODY	52.33	26.36	1	99	−0.303	−0.848
CELF O.C	35.05	26.34	1	98	0.730	−0.394
CELF Morph.	68.58	21.80	2	98	−0.859	0.455
IMErr.	4.43	2.87	0	15	1.12	1.749
DMErr.	9.02	3.99	1	22	0.722	1.708
CMErr.	1.04	0.78	0	4	1.476	3.975

Note. PEABODY = receptive vocabulary percentile score; CELF O.C = oral comprehension percentile score; CELF Morph. = morphological ability percentile score; IMErr. = raw score of inflectional morphology errors in Oral Morphological Awareness (OMA) task; DMErr. = raw score of derivational morphology errors in OMA task; CMErr. = raw score of compounding morphology errors in OMA task.

**Table 2:** Inter-subject effects of MANCOVA for morphological errors in the OMA task by group.

Dependent Variable	Group	*M*	*SD*	*F* (1, 89)	*p*	η_p_^2^
IMErr	Control	3.82	1.78	29.46	<0.001	0.249
	At risk	5.11	3.64			
DMErr	Control	8.80	2.62	24.53	<0.001	0.216
	At risk	9.27	5.13			
CMErr	Control	1.04	0.46	13.59	<0.001	0.132
	At risk	1.05	1.03			

Note. IMErr = inflectional morphology errors in OMA task; DMErr = derivational morphology errors in OMA task; CMErr = compounding morphology errors in OMA task.

**Table 3:** Inter-subject effects of MANCOVA for morphological errors in the OMA task by cycle.

Dependent Variable	Group	*M*	*SD*	*F* (1, 89)	*p*	η_p_^2^
IMErr	First cycle	4.67	3.06	23.16	<0.001	0.206
	Higher cycles	4.08	2.58			
DMErr	First cycle	9.93	3.81	32.68	<0.001	0.269
	Higher cycles	7.71	3.94			
CMErr	First cycle	1.22	0.79	21.25	<0.001	0.193
	Higher cycles	0.79	0.70			

Note. IMErr: inflectional morphology errors in OMA task; DMErr: derivational morphology errors in OMA task; CMErr: compounding morphology errors in OMA task.

## Data Availability

The data supporting this study are not publicly available due to ethical and privacy restrictions.
